# Acceptability and feasibility of recruitment and data collection in a field study of hospital nurses’ handoffs using mobile devices

**DOI:** 10.1186/s40814-018-0353-x

**Published:** 2018-10-24

**Authors:** Patrick Lavoie, Sean P Clarke, Christina Clausen, Margaret Purden, Jessica Emed, Tanya Mailhot, Valerie Frunchak

**Affiliations:** 1 William F. Connell School of Nursing, Boston College, Chestnut Hill, MA USA; 2 Faculty of Nursing, Pavillon Marguerite d’Youville, C.P. 6128 succ. Centre-ville, Montreal, QC H3C 3J7 Canada; 3 Center for Nursing Research, Jewish General Hospital, Montreal, Canada; 4 Ingram School of Nursing, McGill University, Montreal, Canada; 5 Department of Nursing, Jewish General Hospital, Montreal, Canada; 6 Montreal Heart Institute, Montreal, Canada

**Keywords:** Mobile devices, Handoff, Nursing, Acceptability, Feasibility, Research procedure, Recruitment

## Abstract

**Background:**

The portability and multiple functionalities of mobile devices make them well suited for collecting field data for naturalistic research, which is often beset with complexities in recruitment and logistics. This paper describes the implementation of a research protocol using mobile devices to study nurses’ exchanges of patient information at change of shift.

**Methods:**

Nurses from three medical and surgical units of an acute care teaching hospital in Montreal, Canada, were invited to participate. On 10 selected days, participants were asked to record their handoffs using mobile devices and to complete paper questionnaires regarding these exchanges. Nurse acceptance of mobile devices was assessed using a 30-item technology acceptance questionnaire and focus group interviews. The principal feasibility indicator was whether or not 80 complete handoffs could be collected on each unit.

**Results:**

From October to December 2017, 63 of 108 eligible nurses completed the study. Results suggest that the use of mobile devices was acceptable to nurses, who felt that the devices were easy to use but did not improve their job performance. The principal feasibility criterion was met, with complete data collected for 176, 84, and 170 of the eligible handoffs on each unit (81% of eligible handoffs). The research protocol was acceptable to nurses, who felt the study’s demands did not interfere with their clinical work.

**Conclusions:**

The research protocol involving mobile devices was feasible and acceptable to nurses. Nurses felt the research protocol, including the use of mobile devices, required minimal investment of time and effort. This suggests that their decision to participate in research involving mobile devices was based on their perception that the study protocol and the use of the device would not be demanding. Further work is needed to determine if studies involving more sophisticated and possibly more demanding technology would be equally feasible and acceptable to nurses.

**Electronic supplementary material:**

The online version of this article (10.1186/s40814-018-0353-x) contains supplementary material, which is available to authorized users.

## Background

Handheld mobile devices are now widely accessible and powerful enough to accomplish many of the same functions as desktop computers. In healthcare, mobile devices are used for a variety of purposes, including communication, information, patient management, and education [1]. There are published studies on the implementation and evaluation of mobile devices in the delivery of healthcare [2], but there is sparse literature regarding use of mobile devices in health services research.

One aspect of care delivery that is potentially well suited to the use of mobile devices for data collection is handoff—or handover—research. A handoff is “the exchange between health professionals of information about a patient accompanying either a transfer of control over, or of responsibility for, the patient” [3]. Handoff is only one example of a healthcare communication process that is vulnerable to errors that could lead to serious adverse events. Problems with handoffs have long been recognized as important patient safety issues and contributors to healthcare errors [4]. In the context of nursing care, handoffs occur at every change of shift. One nurse presents details regarding one or more patients to the nurse colleague who will oversee the patient’s or patients’ care next. Ultimately, a hospitalized patient’s nursing care is handed off at least two or three times daily.

There are a number of complexities in studies of nursing handoffs. All involved parties must consent to information being collected and analyzed. It is also difficult to predict which nurses will hand off which patients to which nurses and at what time, given that nurses’ schedules are irregular, especially because many nurses rotate shifts, and given that patient assignments are continuously adjusted. In earlier studies of handoffs, most of the time handoffs, patients, and/or nurses have been sampled without a clear indication of how sample sizes were determined. Recruitment procedures have often been poorly described, and it is thus unclear whether the final samples resulted from careful planning or by chance. Under such circumstances, there is always a risk of inadequate statistical power or sampling biases.

Some earlier research on handoffs has used “real-world” approaches (e.g., [5–10]) executed in open environments that are influenced by many external factors and where researchers have little control over exactly what events will be available for study as opposed to laboratory studies [11]. The goal of this type of naturalistic inquiry is to gain an accurate and representative picture of a phenomenon as it unfolds in context, with minimal—although inevitable—influence from the researchers. For this reason, it is potentially desirable to bring data collection methods to participants in the field and mobile devices emerge as an option for data collection in real-world studies because of their portability. Furthermore, the functionalities of mobile devices are compatible with the most common data collection methods in real-world handoff studies—interviews, surveys, and observations [12]. However, an extensive search of the literature revealed no handoff studies using data collected from mobile devices.

### Objectives

The purpose of this paper is to report on the feasibility and acceptability of a research protocol for collecting nurse-to-nurse handoff data using mobile devices. The research questions were:

- Was the research protocol feasible, i.e., was it possible to execute the recruitment and data collection procedures as planned [13]?
- Was the research protocol acceptable, i.e., was the data collection procedure suitable from the perspective of the nurse participants [13]?
- Were mobile devices acceptable to nurses as a method for collecting handoff data?

## Methods

### Design

**Fig. 1:**
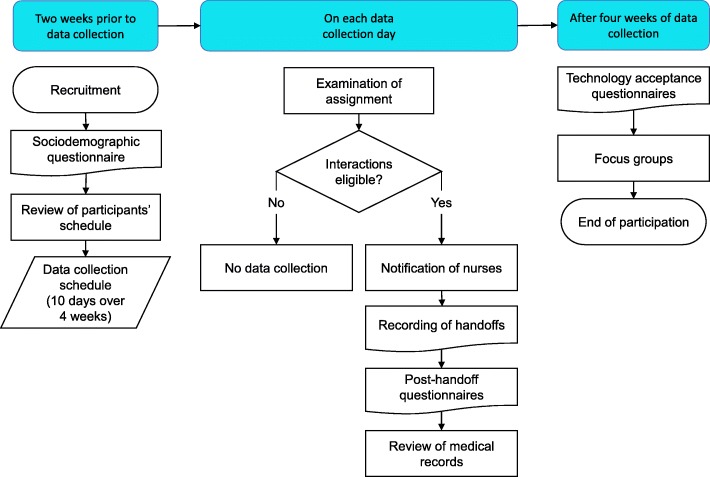
Study procedure flow diagram

This was a convergent mixed-methods [14] feasibility and acceptability study. This study was approved by the institution’s Research Ethics Committee. A flow diagram of the study procedure is presented in Fig. 1.

### Setting

The study was conducted at a tertiary acute care bilingual (English-French) university-affiliated hospital in Montreal, Canada. The purpose of the research protocol was to understand how nurses giving handoffs communicate their judgments of patient risk of deterioration (i.e., their sense that a patient might go into cardiac arrest or be transferred to a critical care unit within the next 24 h). Three non-critical care units specializing in adult surgical (A) and medical (B and C) care were selected because of their high levels of patient acuity and associated high risk of deterioration in the population served. All nurses involved in handoffs on these units were invited to participate in the study. To be eligible for the study, nurses needed to be on duty at least twice during the data collection period.

All units had a similar handoff procedure where the nurse finishing a shift (the outgoing nurse) reports directly to the nurse beginning a shift (the incoming nurse). On the surgical unit (A), nurses work 8-h shifts and patients are handed off three times a day (7:30, 15:30, and 23:30). On medical units (B and C), most nurses work 12-h shifts. This meant that over a 24-h period, patients on units B and C could be handed off between two to four times (7:30, 15:30, 19:30, and 23:30).

### Research protocol

#### Recruitment

Two weeks prior to data collection, nurses’ work schedules were reviewed, and eligible participants were invited in groups to 10-min presentations at their unit nursing stations. Presentations were scheduled to reach nursing staff on every shift and were organized with the help of unit managers. Nurses who were not able to attend the group presentations received one-on-one briefings from the principal investigator (PI) during work hours.

After the presentations, nurses were provided with a card to record their contact information and to drop in a sealed box on each unit if they were interested in participating. The research team followed up with emails to prospective participants to schedule individual meetings during their work hours. Prior to the meetings, potential participants received an electronic copy of the consent form, which the PI then reviewed with participants to answer questions. Participants were encouraged to take as much time as they wanted to decide whether they wanted to participate.

Upon signature of the consent form, participants completed a sociodemographic questionnaire consisting of questions regarding age, gender, primary language, work status (full-time or part-time), nursing experience, and highest nursing degree completed. They received a 5-min training session on how to use the mobile device (demonstration by the PI followed by an exercise for participant to manipulate the device) and were given an advance look at the study questionnaires that would be used in data collection. Upon enrolment in the study, participants received a 10.00 C$ gift card as a token of appreciation.

#### Mobile devices

The research protocol involved recording nurses’ handoffs and collecting survey data. The study used an older generation iPod Touch model marketed between 2010 and 2012 that had been purchased by the hospital for an earlier study. However, the devices were no longer supported by the manufacturer nor were they compatible with most current apps. While a data collection app compatible with the device was identified that would have allowed both recording of handoffs and online completion of study questionnaires, the app employed cloud storage via Wi-Fi. It was discovered during a test run that the hospital’s Wi-Fi bandwidth could not support the volume of data to be transmitted for our study, and we therefore abandoned the idea of using the same app for both recording and survey data collection.

To record handoffs, we selected “Voice Memos,” a start/stop audio recording app developed by Apple. The devices were stripped to make “Voice Memos” the only app available to participants. Audio recordings of handoffs were stored on the devices and transferred to a secured computer using a “hardwired” connection using a data sync cable. Participants completed paper questionnaires, and their responses were entered manually in a database by research assistants.

#### Questionnaire

The questionnaire used for the study incorporated three previously published instruments [5, 15, 16]. There were two versions of the questionnaire tailored to outgoing versus incoming nurses. In both cases, participants were asked to provide (1) their judgment of each patient’s risk of deterioration on a 7-point scale (patient acuity rating) [16], (2) their experience of the interaction with an instrument that was used in a previous study of nursing handoff (seven items for outgoing, nine items for incoming) [5], and (3) their perceived cognitive load (the effort or burden on their working memory) during the interaction on a 9-point scale [15]. Participants rated the risk of deterioration for every patient they handed off; they also rated their experience of the interaction and their cognitive load for each interaction (set of handoffs).

#### Data collection

On each unit, 10 days of data collection were selected over four consecutive weeks, based on the days when the highest numbers of participants were on duty. Participants were asked to provide handoff data on every data collection day and therefore may have been asked to provide data on up to ten shifts over the data collection period. For this study, a “handoff” was defined as the exchange of information about a single patient, and an “interaction” was defined as the exchange of a series of handoffs in a nursing dyad; thus, an interaction could include more than one handoff.

On selected days, the research team examined the nurse-to-patient assignment sheet to identify all interactions to take place at the upcoming change of shift. Nurses were asked to record a handoff if both participants in an interaction were enrolled in the study; if a participating nurse interacted with a non-participating nurse, no recording occurred. No attempts were made to shift the patient assignment of participating nurses, and at no time was a patient reassigned in order to make a handoff eligible for recording.

Approximately 30 min before the selected shift was to begin, the research team informed outgoing nurse participants that their handoffs were to be recorded. These nurses were handed a mobile device and a paper questionnaire. Incoming nurse participants received similar information and were given a similar questionnaire when they arrived to receive their handoffs.

Participants sat together at the nursing station, placed the mobile device between them on a table, and started recording. The nurses themselves were responsible for operating the devices. Immediately afterwards, nurses completed the post-handoff questionnaires. Once nurses handed back their questionnaires and mobile device, the research team examined medical records to collect a set of pre-specified clinical data elements for each patient who had been handed off.

### Feasibility of the research protocol

The principal indicator of feasibility of the protocol was the number of handoffs for which there was complete data, including a recording and two post-handoff questionnaires (outgoing and incoming nurses), as well as a complete set of parameters (systolic blood pressure, heart rate, respiratory rate, temperature, and level of consciousness) needed to calculate a Modified Early Warning Score [17] for the patient. The MEWS is a validated tool used to gauge patient risk of deterioration objectively. Although there is no universally accepted definition of the number of handoffs needed to achieve reliability, we based our target number of handoffs on the numbers collected in earlier published research [7, 8] and decided to aim for at least 60–80 handoffs on each unit.

To further examine the feasibility of the recruitment protocol, we examined the number of eligible nurses who were approached, recruited, enrolled, and who completed the study. For the data collection procedure, we compared the number of eligible handoffs during the study period to the number of handoffs recorded and to the number of handoffs for which we had complete data. We examined rates of and reasons for missing data for handoff recordings, sociodemographic questionnaires, post-handoff questionnaires, and patient data. We also documented the number of distinct patients who appeared in the dataset and the number of times they were handed off. We also documented the average number of handoffs that nurse participants provided.

### End-of-protocol focus groups on acceptability of the research protocol

At the end of the study, all participants were invited to 45-min focus groups after night shifts or on lunch breaks in an office on the units. In total, 16 focus groups were organized with 2.7 (± 1.2) nurses on average. Participants (*n* = 44) were invited to comment on the acceptability of the research protocol, i.e., their perception of its suitability [13]. Using a semi-structured interview guide (Additional file 1), facilitators asked nurses to describe their experience of the data collection procedure, including use of a mobile device for a research study, recording their handoffs, and completing a post-handoff survey. Nurses were asked to identify the strengths and weaknesses of the protocol and to describe what they had learned—if anything—from participating in the study. All focus group interviews were audio recorded.

Focus groups were facilitated by CC, JE, or LC (research assistant). Facilitators had taken formal coursework in qualitative research methods and had various levels of experience with focus group interviews. CC and JE were involved in the design of the study but did not interact with participants during data collection; they were both involved in quality management and research at the hospital where the study was conducted. LC was involved in each step of data collection as a research assistant but had not interacted with participants prior to the study. PL, the PI, did not attend focus groups to allow participants’ a freer environment to express their true feelings and perceptions.

### End of protocol survey on nurses’ acceptance of mobile devices

In addition to the focus groups, all nurses were asked to complete a technology acceptance questionnaire regarding their use of mobile devices to record their handoffs (Additional file 2). The questionnaire was based on the Unified Theory of Acceptance and Use of Technology model (UTAUT) [18]. The UTAUT proposes that users’ intention to use a technological-based device or system is determined by three constructs: performance expectancy (belief that using a system will help to increase job performance), effort expectancy (ease in using the system), and social influence (an individual’s perception that others believe he or she should use a system). The theory posits that actual use of a system is determined by users’ intentions and by facilitating conditions (belief that an infrastructure exists to support the use of the system).

For the present study, we modified the original UTAUT questionnaire to include only the items relevant to the context of this study, for example, a performance expectancy item regarding perceptions of increased chances of getting a raise by using technology was removed. We added four items related to training from a previous study of technology acceptance in hospitals [19] to the modified UTAUT questionnaire. In the end, the nurses were invited to rate their agreement with 30 items on a 7-point scale. The questionnaire contained questions addressing the following constructs: performance expectancy (3 items), effort expectancy (4 items), social influence (4 items), facilitating conditions (3 items), attitude toward using technology (4 items), self-efficacy (3 items), anxiety (4 items), training (4 items), and intention to use (1 item). For each of the nine constructs of the technology acceptance questionnaire, we computed individual scores by calculating means of each participant’s answers on all items for the construct. Individual construct scores were averaged across all subjects.

### Analysis

Data are reported as counts (percentages) for categorical variables and as means ± standard deviations or median (range) for continuous variables. Scores on the technology acceptance questionnaires were compared based on specialties (surgery vs. medicine), work status (full-time vs. part-time), and nursing degree completed (diploma vs. university degree) with independent samples *t* tests. Pearson product-moment correlation was computed to assess the relationship of technology acceptance scores with years of nursing experience and years of experience on the current unit. Statistics were computed using IBM SPSS Version 24.

Focus group data were transcribed and subjected to thematic analysis [20]. Each transcript was read, and meaningful units were identified and coded into categories related to nurses’ experience of the data collection procedure. In each category, codes were combined to create themes that reflected participants’ views. Throughout the analytic process, an audit trail was kept. The analysis was conducted by two researchers who did not participate in the focus groups interviews (PL, TM). They verified and challenged each other’s interpretation of the data. Coding and themes resulting from the thematic analysis were presented to the focus group facilitators for validation, who confirmed that the themes reflected what participants had said during the interviews. Focus group data were managed in Microsoft Word for Mac (version 16.16).

## Results

### Feasibility of the recruitment procedure

**Fig. 2:**
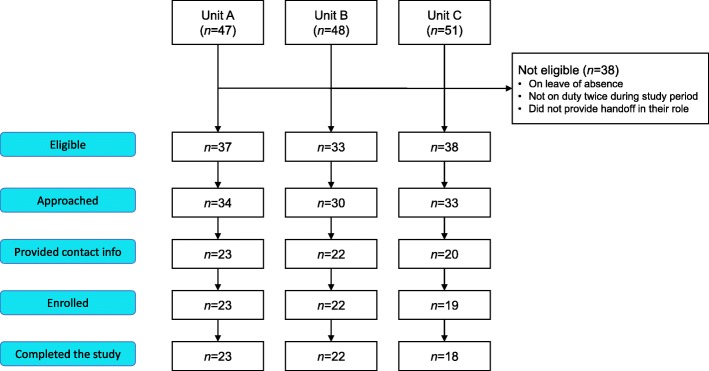
Participant flow diagram

Figure 2 outlines the screening, recruitment, and retention process. From October to December 2017, 146 nurses worked on units A, B, and C. A total of 108 (74.0%) of the nurses were eligible for the study; the remaining 38 nurses were on study and maternity or sick leave, worked part-time, or were in a role where they did not give or receive handoffs.

The study was explained to 97 (90.0%) of the eligible participants. In chronological order, there were nine group presentations on unit A, one group presentation on unit B, and three group presentations on unit C. Our experience with the surgical unit (A) showed that the organization of presentations to groups during work shifts was difficult since nurses were often busy and not able to attend. Thus, one-on-one presentations became the main strategy on the medical units (B and C).

After being informed about the study, 65 nurses (67.0% of those eligible) provided contact information. One nurse who provided contact information decided not to enroll for personal reasons related to being unwilling to take on additional burdens because of family issues. Using the numbers of eligible nurses on the units as denominators, the recruitment rates were 64.9%, 65.7%, and 50.0% on units A, B, and C, respectively. All but one of the enrolled participants completed the study after providing consent: the one participant who withdrew from the study explained that she did so because she believed that rating the likelihood of cardiac arrest or ICU could influence (or “jinx”) the patients’ outcomes. Participants’ sociodemographic characteristics are presented in Table 1.

**Table 1:** Participant sociodemographic characteristics (*N* = 63)

	Statistic
Age (years)^a^	30.8 (8.5)
Gender (female)^b^	51 (81.0)
First language^b^	
English	28 (44.4)
French	24 (38.1)
Other	11 (17.5)
Full-time^b^	35 (55.6)
Nursing experience^a^	4.9 (5.5)
Experience on unit^a^	3.6 (4.1)
Highest degree^b^	
Diploma	17 (27.0)
Bachelor’s	42 (66.7)
Master’s	4 (6.3)

^a^Means with standard deviations

^b^Numbers of participants with percentages

### Feasibility of the data collection procedure

A total of 430 handoffs constituted the final sample obtained. Reasons for losses of eligible handoffs are outlined in Table 2. On the data collection days, 187, 231, and 113 handoffs were eligible for recording on units A, B, and C, respectively. We recorded 180 (96.3%), 188 (81.4%), and 88 (77.9%) of those handoffs. Some handoffs (*n* = 39) were not recorded because they occurred before or after research team members were on the unit or because the number of interactions overwhelmed the team’s ability to distribute and track the devices. On 34 other occasions, nurses accidentally clicked twice instead of once on the “recording” button, thereby stopping the recording, and in two further instances, the recorded files were not retrievable from the device.

**Table 2:** Completion rates

	A	B	C	Total
Eligible handoffs	187	231	113	531
Missed due to research team issues	5	18	16	39
Missed due to device manipulation problems	2	25	9	36
Vital signs	175	181	84	440
Post-handoff questionnaire (outgoing)	175	188	88	451
Post-handoff questionnaire (incoming)	178	183	88	449
Handoffs with complete data	170 (90.9%)	176 (76.2%)	84 (74.3%)	430 (81.0%)

Data are numbers of observations

The patient acuity rating was missing from 12 post-handoff questionnaires. Responses to the questions about the experience of the interactions and cognitive load were missing from one questionnaire where the participant completed only one side of the two-sided form. For a record of a handoff to be complete for the purposes of the study, we required specific patient data elements from medical records. No vital signs were recorded on the shift preceding handoff on four occasions, one or more vital signs were missing on three, and medical records were inaccessible for 11 of the handoffs. Thus, we obtained complete data for 170, 176, and 84 handoffs on units A, B, and C, respectively; the data collection procedure was thus successful for 90.9%, 76.2%, and 74.3% of eligible handoffs on each unit.

Table 3 details the characteristics of the final sample of successfully recorded handoffs for which there were complete data. On the surgical unit (A), most handoffs were recorded at the end of day (15:30) and evening (23:30) shifts. On the medical units (B and C), most handoffs were recorded at the change of 12-h shifts, at 7:30 and 19:30. Across the units, handoffs for 51, 63, and 38 distinct patients were included in the dataset. On average, patients were handed off 3.3, 2.8, and 2.2 times on the respective units. During each nurse-to-nurse interaction, nurses handed off 3.3, 2.5, and 1.8 patients, on average. The number of handoffs per nurse varied greatly across the three units, with an overall mean of 13.9 (± 8.9) handoffs per nurse.

**Table 3:** Characteristics of successfully recorded handoffs

	A (*n* = 170)	B (*n* = 176)	C (*n* = 84)	Total (*n* = 430)
Time recorded (*n*)				
7:30	22	93	46	161
15:30	76	11	0	87
19:30	–	72	38	110
23:30	72	–	–	72
Handoff per patient^a^	3 (1–10)	2 (1–10)	2 (1–6)	2 (1–10)
Handoff per nurse^a^	16 (0–31)	15 (4–46)	7.5 (3–19)	12 (0–46)
As outgoing	8 (0–15)	7 (0–27)	3.5 (0–13)	6 (0–27)
As incoming	6 (0–22)	7 (1–19)	4.5 (0–12)	5 (0–22)

^a^Medians with range

### Acceptability of the research protocol

The thematic analysis revealed that participants were positive about the study. Participants reported that the data collection was not burdensome, did not take too much time, did not delay their work, and did not add too much paperwork. They felt the study was organized: the procedure was very clear and the research team had “taken the guesswork out.” Regarding recruitment, participants had the impression that nurses with fewer years of clinical experience were more interested in participating in the study. They felt that nurses who participated were those who gave better handoffs, were motivated and invested in their nursing practice, and were open to feedback and improvement. They assumed that people chose not to participate if they thought that the study would add to their existing workload, were less receptive to feedback, and/or were more resistant to change.

In terms of their experiences of recording their handoffs, participants’ accounts revealed a tension between a feeling of being evaluated and a desire to “act naturally” while giving handoffs. Because they were being recorded, some participants wanted to sound professional and to give a better report than usual. Others said that they were more explicit in explaining their thought processes, questions, or comments to ensure that their behaviors were caught on tape and examined as part of the study. Other participants wanted to “act natural” and gave handoffs as they usually do. However, participants described how they forgot the recorder over time and became more natural in their handoffs. Some participants even described how they purposely hid the recorder to forget about it and act more “natural.”

According to participants, the content and the structure of the handoffs did not change over the course of the study. They exchanged the same information in the same format, guided by a care planning tool (a generic list of body systems and functions) that nurses used as part of their usual care routines. However, nurses acknowledged that handoffs during the study were more detailed and comprehensive, especially for patients to whom they had previously been assigned and already knew or who had been on the unit for a long time. For some participants, this increased the length of handoffs they contributed to the study. In addition, when nurses were being recorded, they believed they were more mindful of what they were saying and how they were saying it. They reported refraining from behaviors they believed could make them look less professional such as cursing, flamboyant language, jokes, personal opinions, judgmental comments, or personal/friendly interactions with colleagues. As a consequence, some participants felt the study handoffs were shorter because they were more concise, straightforward, and focused. An intriguing finding was that participants discussed these changes with respect to their colleagues’ handoffs but felt their own handoffs did not change.

Participants expressed no concerns about the time to complete the post-handoff questionnaires—they recalled that it took less than 2 min. However, they questioned the questionnaire content. While some nurses felt that it was easy to rate patient risk of deterioration with the information they received during handoff, others felt it was difficult—if not nearly impossible—to predict what would happen to the patient. Others felt that some questions regarding their experience of the interaction did not relate to handoff in any way and were not relevant to the object of study (e.g., feeling emotionally drained, being energized about the shift ahead, feeling a positive connection with the other nurse). They criticized some questions as being too wordy and phrased in the negative. Most participants felt that they did not have a clear sense of “mental effort” being asked about in the cognitive load scale questions and felt a definition of this concept was needed.

Nevertheless, participants acknowledged that being part of the study raised their awareness of the importance of handoff in nursing practice. Some described becoming more reflective regarding their own handoffs and regarding others’ handoffs (e.g., What makes a handoff effective? What information should be shared and emphasized? What information should be left out? How can it be more structured?). Furthermore, being questioned about the relational aspect of handoff made some participants realize that their relationships with colleagues could influence their handoff experiences.

### Nurses’ acceptance of mobile devices

A total of 49 participants (77.8%) completed the technology acceptance questionnaire, and results were similar across units (see Table 4). In the current sample, the modified questionnaire yielded a Cronbach’s alpha of 0.89. Based on a maximum score of seven, participants strongly agreed that the mobile device was easy to use (6.8 ± 0.4); that they had the resources, knowledge, and assistance necessary to use it (6.4 ± 0.9); and that the training they received was satisfactory (6.6 ± 0.7). They somewhat agreed that using the device was enjoyable (4.8 ± 1.2) and that they would use it again (4.9 ± 1.7). They were neutral in their assessments of the influence of colleagues and nursing management regarding use of the device for the study (4.2 ± 1.2). They somewhat disagreed that the device increased their job performance (3.3 ± 1.2). Their self-efficacy and anxiety in using the device were moderate (5.4 ± 1.2) and low (2.0 ± 1.0), respectively.

**Table 4:** Technology acceptance questionnaire subscale scores (*N* = 49)

	Mean (SD)
Performance expectancy	3.3 (1.2)
Effort expectancy	6.8 (0.4)
Social influence	4.2 (1.2)
Facilitating conditions	6.3 (0.9)
Attitude toward using technology	4.8 (1.2)
Self-efficacy	5.4 (1.2)
Anxiety	2.0 (1.0)
Training	6.6 (0.7)
Intention to use	4.9 (1.7)

Comparison of score across nurse characteristics such as specialty, work status, and nursing degree completed revealed no statistically significant differences on most variables. We report the following differences for descriptive purposes, but it is important to note that they were marginal (approaching a *p* < 0.05 level)—adjusting the statistical significance threshold for multiple tests would have rendered each non-significant. Surgical nurses showed higher scores on the attitude toward technology subscale than medical nurses (*M* = 5.3, SD = 1.2 vs. *M* = 4.5, SD = 1.1; *t*(47) = 2.54, *p* = 0.014). Nurses working full-time had lower acceptance scores than nurses working part-time on the following variables: effort expectancy (full-time: *M* = 6.6, SD = 0.5; part-time: *M* = 6.9, SD = 0.3; *t*(46) = − 2.7, *p* = 0.009), facilitating conditions (full-time: *M* = 6.0, SD = 1.0; part-time: *M* = 6.6, SD = 0.7; *t*(47) = − 2.5, *p* = 0.015), and intention to use (full-time: *M* = 4.4, SD = 1.7; part-time: *M* = 5.5, SD = 1.4; *t*(47) = − 2.4, *p* = 0.019). Nurses with university degrees (*M* = 6.5, SD = 0.8) had higher scores on the perception of facilitating conditions than nurses with diplomas (*M* = 5.8, SD = 1.2; *t*(47) = 2.2, *p* = 0.032).

Examination of the relationship between technology acceptance scores and years of experience on the current unit revealed that nurses with more experience perceived that the mobile device was more difficult to use (*r*(48) = − 0.50, *p* < 0.001). They also had lower scores on the perception of facilitating conditions than nurses with less experience on the units (*r*(49) = − 0.43, *p* = 0.002). Correlations between experience on the current unit and scores on the remaining technology acceptance subscales did not reach statistical significance. Since the correlation between nursing experience and experience on current unit was high (*r*(49) = 0.86, *p* < 0.001), results for nursing experience were nearly identical.

In the focus groups, participants reported that using the mobile device was easy, straightforward, and intuitive. The interactions with the device were minimal and not time-consuming. Most participants knew how to operate the device, and if not, they felt they received proper training and that the research team was available for support. Participants also believed that younger nurses were less intimidated by mobile devices. Of note is one participant’s comment that she felt that using a mobile device to collect data was a desirable technological advancement since she perceived that her unit was less technologically advanced than other units in the hospital.

## Discussion

This paper presents an assessment of the feasibility and acceptability of a research protocol to collect nursing handoff data using mobile devices on acute medical and surgical units. Results show that the research protocol was feasible—the target of 60–80 handoffs was met on each unit—and was acceptable to nurses. Additionally, these results give insights into the factors that played in the success of the recruitment and the data collection procedures. Moreover, they provide initial data regarding nurses’ acceptance of mobile devices for research purposes.

One of the main challenges of this study was recruiting a sufficient number of nurses to ensure a robust database of handoffs and related data. Overall, the recruitment rate was equal to or slightly higher than the average recruitment rate in studies involving nurses, which typically fall below 50–60% [21, 22]. One factor consistently found to affect nurses’ participation in research is perceived demands on time [23–25]. In their clinical work, nurses face fitting study-related activities into schedules characterized by multiple competing demands [23]. In the focus groups, participants acknowledged that the data collection procedure did not take too much of their time and did not delay their regular work. Therefore, it would appear that in this study, nurses perceived the investment of time being asked of them as minimal and felt that the data collection procedure could be easily integrated into their regular work routines. Moreover, nurses felt the mobile device/app was easy to use, which suggests that the effort required to use it was minimal. Previous studies have suggested that beyond time and effort requirements, nurses also take the value or the relevance of a study into account when deciding whether to participate in research [24, 26, 27]. Participants stated that the study was interesting and relevant to their clinical work. They also acknowledged that being in the study made them aware of the importance of nursing handoffs. Thus, it seems that the cost-benefit ratio—in terms of time commitment versus value of participating in the study—was favorable. This could have facilitated recruitment and retention in the study, along with other factors that have been previously shown to enhance nurses’ participation in research, such as management/institutional support [27] and monetary incentives or compensation [24].

The sociodemographic characteristics of the study population overall also seemed to have facilitated recruitment. In the focus groups, participants believed that the study attracted younger nurses with fewer years of clinical experience. This was consistent with the participant sociodemographic data showing that participants were approximately 10 years younger than the average age of nurses across Quebec of 41.6 years [28]. Moreover, it seems that many participants had less than 5 years of clinical experience on average (*M* = 4.9, SD = 5.5). Previous studies have suggested that nurses’ participation and interest in research may be negatively correlated with years in practice [26, 29]. A positive relationship between higher education and nurses’ attitudes toward research has also been reported previously [29], and indeed, the majority of this study’s participants held university degrees. On the technology acceptance questionnaire, it was interesting to see that experience (on the current unit or in nursing in general) had a similar effect on nurses’ acceptance of mobile devices. Although other differences on the technology acceptance questionnaire were minimal and have to be considered with caution, the results also suggest that participants’ acceptance of mobile device varied depending on specialty (medical or surgical nursing), education, and work status. However, these findings need to be replicated in a study that is sufficiently powered to control for the influence of other confounding variables.

It was difficult to predict how many handoffs would be eligible for recording, mostly because of nurses’ irregular schedules and varying nurse-to-patient assignments. We found wide variations across units in the number of handoffs that were eligible for recording. This could be attributed to the number of interactions that nurses were involved in at each change of shift. For example, on unit B, nurses handed off 1.8 patients per interaction on average, which means that they were involved in more interactions to handoff the same number of patients. Considering that approximately two out of three nurses were involved in the study, having multiple nurses receiving handoffs from a single outgoing nurse likely reduced the probability that each participating nurse would interact with other participating nurses. More exploration would be required to confirm this, identify any other factors that could affect ability to accumulate handoffs in a timely manner, and identify other ways of optimizing one data collection protocol (for instance, by targeting best times for data collection).

The reasons underlying missing data related mostly to issues in recording. There were proportionately few cases of missing post-handoff questionnaires or patient data. Recording issues were evenly distributed between problems connected to the research team and those associated with participants. Increasing research team coverage of the units (numbers of personnel and hours spent on the units) may have averted some missing data, but given that only 6.8% of eligible handoffs were missed due to issues related to the research team, it may not be worth the cost. Providing more explanations to participations about verifying that the mobile device is recording would appear to be more cost- and time-efficient option to generate more usable data, especially since most training sessions lasted under 2 min.

Our results indicate that placing nurses in control of data collection using mobile devices was effective and well accepted by participants. Overall, the devices were easy to use, and participants were able to operate them—either based on previous experiences or from the training they received in the study. They appear to have seen the devices as a data collection tool like any other but did not perceive any benefits from using the devices. In the end, focus group results suggested that participants were more concerned with being recorded than with the recording devices themselves. Previous studies of health professionals with and without audio recording yielded similar recruitment rates [22, 30], suggesting that willingness to participate was not affected by recording as a data collection method.

How being observed influences behavior is a topic of much discussion in a variety of social science disciplines; self-presentation—people’s attempts to control others’ impressions of themselves—is a known fact of social life [31]. In the present study, nurses felt that recording influenced the length of their handoffs and what they referred to as “less-professional behaviors.” Nonetheless, “less-professional behaviors” were noted in the recordings, suggesting that participants may not have been altering their behaviors as much as they may have thought. Furthermore, participants believed that the content and structure of handoffs remained the same and that initial differences observed diminished over time once they became accustomed to and more comfortable with being recorded. All of this suggests that recordings do, in fact, yield meaningful insights regarding the content and structure of handoffs.

Participants seemed more concerned with the questions on the post-handoff questionnaire than with recording or with the use of mobile devices. Criticisms related to language/phrasing in the questionnaires, such as negative wording, were noted and could be revised in future research. Criticisms related to the pertinence of the questions appeared to be related to participants’ lack of familiarity with the study’s theoretical basis and with the meaning of certain concepts. For example, it was not overtly stated that the study was exploring the relational aspect of handoffs. Consequently, participants questioned why they were asked about how their interactions with their colleagues. We chose a single item to measure participants’ level of mental effort; this question appears to have been poorly understood. Other measures of cognitive load in handoff exist [32], but they are more lengthy and would likely increase participants’ perception of burden. Future studies might test the acceptability of a multi-item scale to measure nurses’ cognitive load during handoffs.

## Conclusions

In this study, the use of mobile devices for handoff data collection was acceptable to nurses. Given their portability and functionalities, mobile devices appear to be feasible tools for real-world handoff studies. The most challenging issues that were experienced during this study related to network capacities and app compatibility, rather than to nurses’ willingness to use the device. However, the app was rather unsophisticated relative to more recent apps that were unfortunately not compatible with the device used for this study. It is unclear how accepting nurses would have been of a more sophisticated app that would have increased the complexity of the efforts asked of them without offering any assistance or benefit to them. The UTAUT model [18] would suggest that this would decrease nurses’ acceptance of mobile devices for data collection. This is a clear design challenge, especially considering that the balance between a study’s demands and nurses’ perception of its relevance and benefits has long been recognized to affect their participation in research. It seems that both recruitment for this study and nurses’ acceptance of mobile devices resulted from a positive balance between perceptions of benefits against perceived costs among those approached, which is a principle that bears considerations in future research in this and similar fields.

## Additional files

Additional file 1: Focus groups interview guide. (DOCX 21 kb)

Additional file 2: Technology acceptance questionnaire. (DOCX 31 kb)

## Abbreviations

MEWS: Modified Early Warning Score
PI: Principal investigator
UTAUT: Unified Theory of Acceptance and Use of Technology
