# Supplementary material for: Acceptability and feasibility of recruitment and data collection in a field study of hospital nurses’ handoffs using mobile devices
Source: Pilot Feasibility Stud. 2018 Oct 24;4:163. doi: 10.1186/s40814-018-0353-x (PMC6199701; doi:10.1186/s40814-018-0353-x)
Supplement: Supplementary file 1 — Focus groups interview guide. (DOCX 21 kb) [file 40814_2018_353_MOESM1_ESM.docx]

**Additional file 1**

**Focus Groups Interview Guide**

**Opening** (5 min): I want to thank you for attending this focus group discussion. You have been invited because you participated in the handoff study. My name is (…) and I am a researcher for this study. I will be facilitating the discussion. I am with (…) who is a research assistant on this study and who will take notes during the discussion.

To keep track of our discussion, I will be recording the discussion. However, our exchanges are strictly confidential. Your names will not appear on the transcript of the discussion nor on any research reports or papers.

Today’s objective is to learn about your experience of the data collection procedure for the handoff study and, more broadly, your experience of nursing handoffs in general. We have prepared questions to facilitate our discussion. Please feel free to discuss your real opinion—there are no right or wrong answers. All your feedback and insights are welcome. Nevertheless, I would ask that you do not use individuals’ names during the discussion. You can refer to them with their roles (e.g. charge nurse, nurse manager, patient, nurse).

Do you have any questions before we start? If you agree, I will start recording now.

1. Data collection procedure (30 min)
   1. Tell us about your experience with the data collection procedure.
      1. Using an app for a research study.
      2. Recording handoffs.
      3. Rating a patient’s risk of deterioration.
      4. Completing a post-handoff questionnaire.
   2. What were the strengths and weaknesses of this data collection procedure?
   3. What did you learn from participating in this study?
2. Handoff experience (30 min)
   1. Tell us about your experience with nursing handoff.
   2. What are the strengths and weaknesses of the nursing handoff procedure on your unit?
   3. What could be done to improve the handoff procedure for your unit?
3. What advice would you give to a researcher who is going to repeat this type of study?
4. Do you have anything else that you would like to discuss before we end this interview? (5 min)
