# Supplementary material for: Acceptability and feasibility of recruitment and data collection in a field study of hospital nurses’ handoffs using mobile devices
Source: Pilot Feasibility Stud. 2018 Oct 24;4:163. doi: 10.1186/s40814-018-0353-x (PMC6199701; doi:10.1186/s40814-018-0353-x)
Supplement: Supplementary file 2 — Technology acceptance questionnaire. (DOCX 31 kb) [file 40814_2018_353_MOESM2_ESM.docx]

**Additional file 2**

**Technology Acceptance Questionnaire**

By completing this questionnaire, we will learn more about your experience of using an iPod to record your handoffs during the Handoff Study. We expect that it will take approximately 15-30 minutes to complete the questionnaire.

**Please use the scale below to describe your agreement with each statement:**

| **1** | **2** | **3** | **4** | **5** | **6** | **7** |
| --- | --- | --- | --- | --- | --- | --- |
| Strongly disagree | Disagree | Somewhat disagree | Neutral | Somewhat agree | Agree | Strongly agree |

| TRAINING | | **1** | **2** | **3** | **4** | **5** | **6** | **7** |
| --- | --- | --- | --- | --- | --- | --- | --- | --- |
| 1 | The content of the training that I received before using the iPod was satisfactory |  |  |  |  |  |  |  |
| 2 | The level of the training that I received before using the iPod was satisfactory |  |  |  |  |  |  |  |
| 3 | The way I was trained before using the iPod was satisfactory |  |  |  |  |  |  |  |
| 4 | Overall, the training I received was sufficient |  |  |  |  |  |  |  |
| EASE OF USE | | **1** | **2** | **3** | **4** | **5** | **6** | **7** |
| 5 | Learning to operate the iPod was easy for me |  |  |  |  |  |  |  |
| 6 | It would be easy for me to become skillful at using the iPod |  |  |  |  |  |  |  |
| 7 | My interaction with the iPod was clear and understandable |  |  |  |  |  |  |  |
| 8 | I believe that the iPod was easy to use |  |  |  |  |  |  |  |
| SOCIAL | | **1** | **2** | **3** | **4** | **5** | **6** | **7** |
| 9 | People who influence my behaviour think that I should use the iPod |  |  |  |  |  |  |  |
| 10 | People who are important to me think that I should use the iPod |  |  |  |  |  |  |  |
| 11 | In general, the organization has supported the use of the iPod |  |  |  |  |  |  |  |
| 12 | The senior management has been helpful in the use of the iPod |  |  |  |  |  |  |  |
| PERFORMANCE | | **1** | **2** | **3** | **4** | **5** | **6** | **7** |
| 13 | Using the iPod increases my productivity |  |  |  |  |  |  |  |
| 14 | I would find the iPod useful in my job |  |  |  |  |  |  |  |
| 15 | Using the system enables me to accomplish tasks more |  |  |  |  |  |  |  |
| SELF-EFFICACY | | **1** | **2** | **3** | **4** | **5** | **6** | **7** |
| 16 | I could use the iPod if there was no one around to tell me what to do |  |  |  |  |  |  |  |
| 17 | I could use the iPod if I could call someone for help if I got stuck |  |  |  |  |  |  |  |
| 18 | I could use the iPod if I had a lot of time |  |  |  |  |  |  |  |
| 19 | I hesitate to use the iPod for fear of making mistakes I cannot correct |  |  |  |  |  |  |  |
| ANXIETY | | **1** | **2** | **3** | **4** | **5** | **6** | **7** |
| 20 | I feel apprehensive about using the iPod |  |  |  |  |  |  |  |
| 21 | It scares me to think that I could lose a lot of information by hitting the wrong button |  |  |  |  |  |  |  |
| 22 | The iPod is somewhat intimidating to me. |  |  |  |  |  |  |  |
| FACILITATING CONDITIONS | | **1** | **2** | **3** | **4** | **5** | **6** | **7** |
| 23 | I have the resources necessary to use the iPod |  |  |  |  |  |  |  |
| 24 | I have the knowledge necessary to use the iPod |  |  |  |  |  |  |  |
| 25 | A specific person (or group) is available for assistance with difficulties |  |  |  |  |  |  |  |
| ATTITUDE | | **1** | **2** | **3** | **4** | **5** | **6** | **7** |
| 26 | The iPod app makes work more interesting |  |  |  |  |  |  |  |
| 27 | Working with the iPod is fun |  |  |  |  |  |  |  |
| 28 | I like working with the iPod |  |  |  |  |  |  |  |
| INTENTION TO USE | | **1** | **2** | **3** | **4** | **5** | **6** | **7** |
| 29 | I would use the iPod again |  |  |  |  |  |  |  |

**Please circle your answer:**

30. Do you think that using the iPod to record your handoffs was a bad or a good idea?

| **1** | **2** | **3** | **4** | **5** | **6** | **7** |
| --- | --- | --- | --- | --- | --- | --- |
| Bad idea |  |  | Neutral |  |  | Good idea |

**Please use this space to add anything you would like us to know:**
